# A simple model for the early events of quorum sensing in *Pseudomonas aeruginosa*: modeling bacterial swarming as the movement of an "activation zone"

**DOI:** 10.1186/1745-6150-4-6

**Published:** 2009-02-12

**Authors:** Sergiu Netotea, Iris Bertani, Laura Steindler, Ádám Kerényi, Vittorio Venturi, Sándor Pongor

**Affiliations:** 1 Biological Research Center of the Hungarian Academy of Sciences, H-6701, Szeged, Temesvári krt. 62, Hungary; 2 International Centre for Genetic Engineering and Biotechnology, 34012 Trieste, Italy

## Abstract

**Background:**

Quorum sensing (QS) is a form of gene regulation based on cell-density that depends on inter-cellular communication. While there are a variety of models for bacterial colony morphology, there is little work linking QS genes to movement in an open system.

**Results:**

The onset of swarming in environmental *P. aeruginosa *PUPa3 was described with a simplified computational model in which cells in random motion communicate via a diffusible signal (representing *N*-acyl homoserine lactones, AHL) as well as diffusible, secreted factors (enzymes, biosurfactans, i.e. "public goods") that regulate the intensity of movement and metabolism in a threshold-dependent manner. As a result, an "activation zone" emerges in which nutrients and other public goods are present in sufficient quantities, and swarming is the spontaneous displacement of this high cell-density zone towards nutrients and/or exogenous signals. The model correctly predicts the behaviour of genomic knockout mutants in which the QS genes responsible either for the synthesis (*lasI, rhlI*) or the sensing (*lasR, rhlR*) of AHL signals were inactivated. For wild type cells the model predicts sustained colony growth that can however be collapsed by the overconsumption of nutrients.

**Conclusion:**

While in more complex models include self-orienting abilities that allow cells to follow concentration gradients of nutrients and chemotactic agents, in this model, displacement towards nutrients or environmental signals is an emergent property of the community that results from the action of a few, well-defined QS genes and their products. Still the model qualitatively describes the salient properties of QS bacteria, i.e. the density-dependent onset of swarming as well as the response to exogenous signals or cues.

**Reviewers:**

This paper was reviewed by Gáspár Jékely, L. Aravind, Eugene V. Koonin and Artem Novozhilov (nominated by Eugene V. Koonin).

## Background

**Figure 1:**
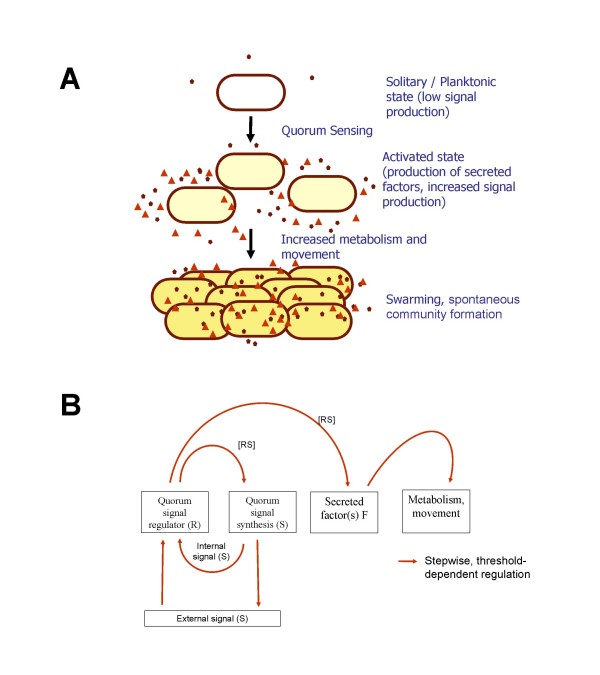
**A) The principle of QS-mediated swarming in *P. aeruginosa***. **B) **Simplified regulatory framework. The system has a single QS signal *S *(that *in vivo *corresponds to C12-3-oxo-AHL and C4-AHL). If the level of *S *exceeds a certain threshold level, the cell becomes activated. Production of *S *is increased by a positive feedback loop, and production of a factor *F *starts. *F *corresponds to all secreted factors (i.e. "public goods") such as surfactants, enzymes, siderophores, and so on, that the cells secrete into the environment. If the concentration of *F *exceeds a threshold, the cells start to swarm: they increase their movement, nutrient intake, and metabolism.

Quorum sensing (QS) is a form of gene regulation based on cell-density, which depends on inter-cellular communication involving the production of and response to signaling molecules [1] (Figure 1A). QS is advantageous to a community of bacteria by facilitating adaptation to changing environmental conditions and enhancing their defense capabilities against other microorganisms or eukaryotic host-defense mechanisms. In Gram-negative bacteria, the most common QS system involves the production and response to an acylated homoserine lactone (AHL). A typical AHL-dependent QS system is mediated by two proteins belonging to the LuxI-LuxR protein families; LuxI-type proteins are responsible for synthesizing AHLs which, in turn, interact directly at quorum concentration with the cognate LuxR-family protein. Subsequently, this complex is able to affect target gene transcription [2]. The QS regulatory network contains a positive feedback loop that is thought to be necessary for its switch-like-behavior as well as for the stability of its 'on' and 'off' states with respect to molecular noise [2-4].

In *Pseudomonas aeruginosa*, the AHL QS circuitry is complex and hierarchical, consisting of two AHL QS systems called LasI/R and RhlI/R. The LasI/R system synthesizes and responds to *N-3*-oxo-dodecanoyl-homoserine lactone (C12-3-oxo-AHL) whereas RhlI/R synthesizes and responds to *N*-butanoyl-homoserine lactone (C4-AHL). These two systems are global, regulating hundreds of genes in *P. aeruginosa*, and playing key roles in colonization and pathogenesis (reviewed in [5]). An important phenotype regulated by AHL QS in *P. aeruginosa *is the swarming motility, which is a community phenomenon involving the fast movement of a bacterial population on a semi-solid, viscous surface [6]. Swarming by *P. aeruginosa *is characterized by a dendritic community appearance and has been shown to require the expression of several loci including flagella, pili, and rhamnolipid-encoding genes [7]. AHL QS is pivotal for the swarming of *P. aeruginosa*, as it is involved in the regulation of many of the genes required for this community behavior (Figure 1; [8,9]). *P. aeruginosa *has been studied intensely, mainly for its ability to opportunistically cause chronic infections in hospitalized and immunocompromised human hosts, and for being the major cause of death in cystic fibrosis patients [10]. Importantly, *P. aeruginosa *is also an efficient colonizer of animal, soil, water, and plant environments.

The first step towards modeling the colonization abilities of *P. aeruginosa *is defining the biological, experimental, and computational frameworks. The biological question we seek to answer is the contribution of QS to the onset of swarming. We define swarming as the concerted movement of a bacterial community in a given direction, for example, towards nutrients and/or other exogenous signals. In accordance with previous molecular studies, we base the model on the regulation of a few key genes. The experimental framework is based on the growth properties of bacteria on agar plates. We used the so-called swarming agar plates that allow the growth of activated bacteria, but not of non-activated ones. The choice of the computational framework is especially critical, since bacterial growth can be modeled with a variety of techniques, most of which are directed to colony morphology. The underlying methods fall into two broad categories (for reviews see [11,12]): **i) ***Continuum models *treat bacterial colonies as a continuous material that diffuses and expands in an environment of other continuous materials in a process described by reaction-diffusion equations [11,12]. Bees et al. used continuum models to describe the colony morphology of the quorum-sensing bacterium *Serratia liquefaciens *[13,14]. **ii) ***Hybrid models *use a continuum description for the growth medium as well as for the solutes, and individual descriptions of bacteria. One of the best known models [11,15,16] is based on cell clusters (groups of cells consisting of up to 10^4 ^cells) that have their own rules for division, growth, and interaction, and orient their movement according to various concentration gradients (nutrients, chemotactic signals, and so on) within the medium. In a recent study, Gerlee and Anderson presented yet a different kind of hybrid model in which individual cells were represented by cellular automata fixed to regularly spaced locations representing the culture medium [17]. A considerable part of computational efforts has been devoted to the simulation of branched colony patterns, which various microbes are known to produce under harsh growth conditions [12,15,18,19]. Even though the formation of bacterial colonies is a complex process that requires a variety of mechanisms (including flagella, pili, secretion of surfactants, siderophores, and enzymes), both continuum models and particle-based hybrid models can reproduce the fractal-like branched patterns characteristic of mature bacterial colonies.

Here we address the onset of quorum sensing-mediated swarming in *P. aeruginosa*. We consider the initial phase of swarming as being controlled by threshold levels of AHL signals and secreted factors (public goods), under the dual control of regulatory proteins and signal synthases of the LasI/R and RhlI/R AHL QS systems. We compare the swarming behavior of the wild-type strain and quorum sensing knock-out mutants in the presence and absence of exogenous AHL signal molecules. We present a simplified, agent-based model for describing the onset of QS, based on a threshold-dependent representation of the early regulatory events and demonstrate that our simplified model provides a qualitatively correct description of the swarming response.

## Results

### Biological model

We compare the behavior of environmentally isolated *P. aeruginosa *PUPa3 and two derivative AHL QS mutants (Table 1). In mutant *P. aeruginosa *SN (signal negative), both the *lasI *and *rhlI N*-acyl homoserine lactone (AHL) synthase genes were inactivated. Thus, strain SN is unable to produce two specific types of signal molecules (C4-AHL and C12-3-oxo-AHL); however, it is expected to respond if these are added to the growth medium. In mutant SB (signal blind) on the other hand, the LuxR family, response-regulator genes *lasR *and *rhlR *were both inactivated, thus SB is unable to respond to the AHL signals. We tested the mutants on swarming agar plates, that is, semi-viscous plates poor in nutrients. These are relatively harsh growth conditions as compared to plates rich in media (Methods). The cells were found to behave as expected according to their genotype (Table 1), and their behavior will be further discussed below, while being compared with the computational model (Figures 5 and 6, further below). Briefly, *P. aeruginosa *cells moved as previously reported, spreading on a swarming plate, accelerating biomass production, in a typical dendritic colonial appearance. Similarly to other strains, AHL QS was pivotal for swarming in *P. aeruginosa *PUPa3, probably through the regulation of several factors, including the RhlI/R-regulated rhamnolipid production. Swarming of *P. aeruginosa *has been associated with both flagella and type IV pili, which mediate the actual movement, as well as with rhamnolipids that allow cells to overcome water's surface tension. The results confirm that QS regulation in the rhizosphere colonizer PUPa3 strain is similar to that seen in clinical isolates of *P. aeruginosa*.

**Table 1:** Genotype and expected phenotype of *P. aeruginosa PUPa3 *and its knock-out mutants

**Cells**	**Genotype**				**Expected phenotype**					
					
					**Signal**		**Factor**		**Swarming**	
	*LasR*	*LasI*	*RhlR*	*RhlI*	Produces	Responds	Produces	Responds	Alone	With added signal

WT	+	+	+	+	+	+	+	+	+	+

SN	+	-	+	-	-	+	+	+	-	+

SB	-	+	-	+	(+)	-	-	+	-	-

### Computational model

We constructed a simplified logical framework that incorporates the salient features of the early regulatory events (Figure 1B, **above**). In this scheme, there is only one signal molecule *S*, which corresponds to C4-AHL and C12-3-oxo-AHL of *P. aeruginosa*. The cellular concentration of this signal is in equilibrium with the environment. *In vivo*, AHL signals activate the synthesis of various secreted factors, such as the biosurfactant rhamnolipid, which is necessary for the cells to move on the surface, enzymes, such as proteases that digest macromolecular nutrients in the environment, antibiotic compounds that fend off competition by other bacteria, siderophores that help collect metal ions from the environment, and so on. In our simplified model, all public goods are included in a generalized secreted factor *F*. This factor is in equilibrium with the environment and stimulates the cells' metabolism and movement. If the concentration of *F *is greater than a given threshold, the cells move and divide faster and consume more nutrients, that is, they initiate swarming. The model has three states: **i) **the solitary or planktonic state, **ii) **the activated state, and **iii) **the swarming state (see **Methods **for more details). In the solitary or planktonic state, cells produce low levels of signal molecules, and have low rates of movement and metabolism. Once the level of *S *reaches a threshold, the cells enter the activated phase in which **a) **the signal production increases and **b) **production of secreted factors ("public goods") starts.

### Algorithmic model

**Figure 2:**
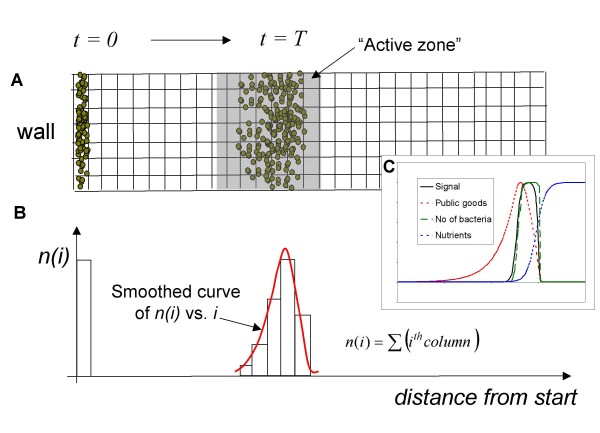
**Model outline**. The model describes the movement of cells on a longitudinal segment of the plane, discretized into squares (**A**). On the longitudinal sides, the track has periodic boundary conditions with respect to cell movement and diffusion. At the beginning (t = 0), the cells are placed at the starting point at random positions. At each time point, the cells carry out the algorithm described in the Methods section (Figure 8). As a result, the cells form an advancing front, and at each time T, the distribution of cell density as well as signal concentration is determined. The distributions found are irregular and asymmetrical (**inset C**).

We chose an agent-based scenario to simulate the movement of cells on a 2D agar surface (see **Methods**). During each time interval, the cells move to a new location, consume nutrients, and produce AHL signals. The cells make steps of equal length in a randomly selected direction, and if they get into a region with insufficient supply of nutrients, they enter a stationary phase. If nutrients are available locally, the cells ingest them in terms of "energy", and if the stored energy exceeds a certain level, they divide. This is a highly simplified scenario, in which the cells do not sense the nutrients' concentration or gradient, and/or the AHL signals, and they do not orient their movements as a function of these gradients. Rather, they simply switch on and off their genes in a threshold-dependent manner. At the beginning of the simulation, the cells are placed at the starting line of a longitudinal track representing the agar plate (Figure 2). In contrast to full colony morphology models, this setting includes only a small portion of the colony. During the simulation, the randomly moving cells spontaneously form a front or "activation zone" in which the level of public goods is sufficient for keeping the cells in an activated state. This zone then spontaneously moves in one direction, i.e., towards the nutrient-rich region. At given intervals during the run, the cell density is calculated by counting the cells within selected areas or the race track. In addition to the moving cell agents, the model includes diffusible materials (nutrients, AHL signals) that are allowed to diffuse at each time point. The density of the cells and the concentration of the AHL signals show irregular bell-shaped curves (Figure 2, **inset**). In the above model system, all quantities are defined in arbitrary units, and only a few "realistic" choices are made. For instance, we assume that AHL signal production substantially increases as the cells become activated [20]. Also, we assume that the production of the AHL signal requires relatively little energy, while the production of factor *F *is much more energy-expensive. This is based on the well-known fact that swarming requires massive quantities of secreted factors such as enzymes, siderophores, and surfactants produced by a large number of genes, as opposed to the relatively few signaling genes [2,5].

### Basic properties of the in silico model

**Figure 3:**
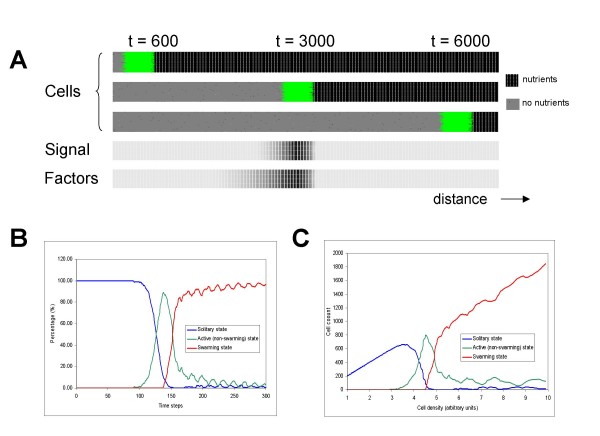
**Model behavior**. **A) **Snapshots of the advancing front. The green area shows cells in the swarming state. The concentration of signal S and factor F follows the movement of bacterial cells. The lower panels show the very first steps of a simulation starting from a very small population (200 solitary cells). **B) **Percentage of solitary (blue), signal-producing but not yet swarming (green) and swarming (red) cells in a population. Note that after a certain time, practically all cells are in the swarming state. **C) **Dependence of the cells' state on cell density. Note that nearly beyond a certain cell density, all cells switch to the swarming state, i.e. the model acts as a density-switch.

Typical simulation snapshots are shown in Figure 3A. The actively swarming cells are shown in green and the active zone, that is, the zone in which the cells are active and swarming, is clearly visible throughout the simulation.

Swarming and non-swarming models show distinctly different pictures in this scenario. Swarming cells form colonies that move relatively fast, the number of cells present in the advancing front increases and reaches a plateau that corresponds to the maximal cell density allowed. On the other hand, non-swarming colonies can not move, so their number can increase only as long as nutrients are locally available. As nutrients are depleted, the number decreases to a baseline level that can be supported by the diffusion of nutrients (not shown). The definition of QS is that cells respond to cell density. Figure 3B and 3C show that the model-population in fact acts as such a density switch. At a given cell density the cells get activated i.e. they start to produce factors (green line), and subsequently the cells also start to swarm (red line). It is worth to note that the starting population is random (both in terms of locations and in terms of metabolic states). Nevertheless, this random population shows a coordinated behavior as it switches from solitary to swarming state.

**Figure 4:**
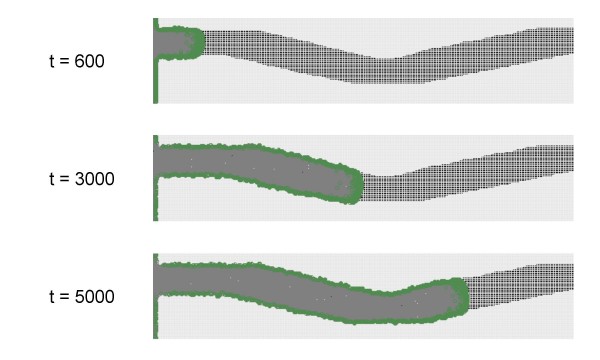
**Activation of cell agents by an exogenous signal**. In this experiment, a non-diffusing and non-decaying signal S was provided in the form of an irregular trail. The cellular agents were of the SN type that are unable to produce the signal but can respond to it. Note that swarming occurs only along the signal trail. (screenshots taken at different times *t*, *t *expressed in arbitrary units).

In the modeling experiments presented so far, the bacterial front followed the availability of nutrients. On the other hand, nutrients and signals are both required for movement, which implies that the cell agents are in principle also capable of following a trail of exogenous signals. The example in Figure 4 illustrates this property of the model. Thus it seems that a simple gene-activation model is sufficient to explain the response of bacteria not only to cell density, but also to exogenous cues such as e are known to play roles in host/symbiont and plant/pathogen interactions.

### Swarming of *P. aeruginosa in vivo *and *in silico*

**Figure 5:**
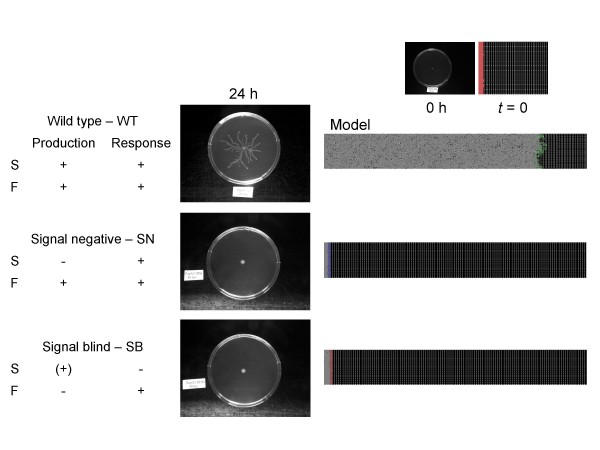
**Swarming of *P. aeruginosa *in vivo and *in silico***. The cells' phenotype is indicated on the left. The swarming plates (center) were developed as described in the Methods section. The computer model (left) shows the behavior of the *in silico *model, which is in agreement with the lab. Green color was used for the wild-type, blue for SN, and red for SB cells. (+) indicates that SB cells have a basal (solitary) level expression of signal S, that is preserved in all states but does not increase upon the onset of swarming.

**Figure 6:**
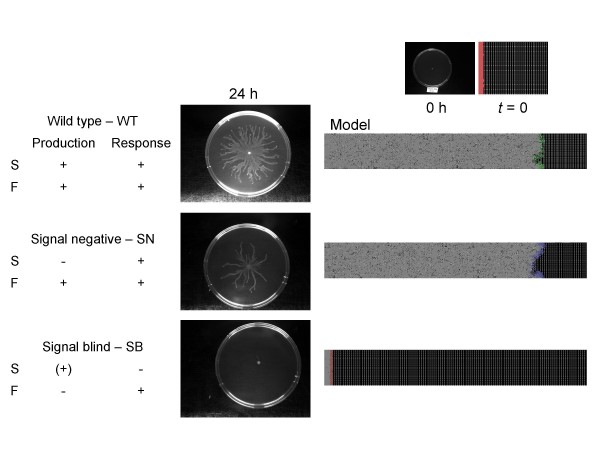
**Swarming in the presence of exogenous signals**. The outline of the experiments is the same as in Figure 4, except that the colonies were left to develop in the presence of exogenous AHL signals. (In the lab experiments, AHL signals were added to swarming plates, to a final concentration of 2 μM, whereas in the computer simulation, the signal concentration was simply kept above the threshold level). (+) indicates that SB cells have a basal (solitary) level expression of signal S, that is preserved in all states but does not increase upon the onset of swarming. Note that SN swarms in response to the signal, while SB does not. The behavior of the wild-type was approximately similar to that observed in Figure 5.

The behavior of wild-type *P. aeruginosa *PUPa3 as well as its mutants is compared in Figures 5, 6. In the absence of exogenous AHL signal, only the wild-type cells swarm (Figure 5). If the exogenous AHL signal is added to the plates, the SN mutants will also swarm, both *in vivo *and *in silico*, yet the SB mutants will not (Figure 6). These results show that **i) **the genetic modifications produced the expected phenotypes (Table 1), and **ii) **the simplified regulatory scheme built into the agent-based model provides a qualitatively adequate description of the events.

### Kinetics and competition *in silico*

**Figure 7:**
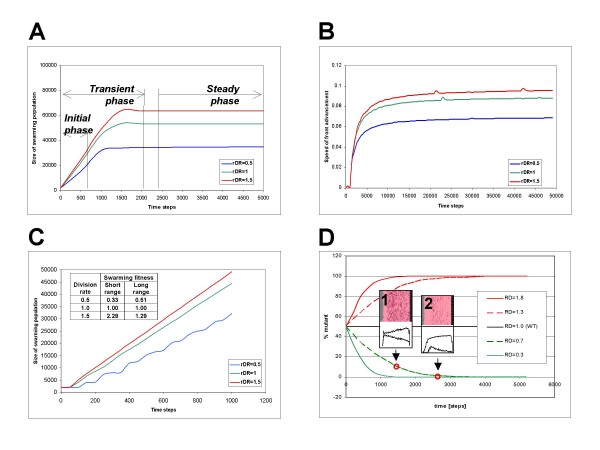
**Simulation of mutants with different rates of division as compared to the wild-type *P. aeruginosa***. The mutant models are similar to the wild type except that the energy expenditure is altered, as compared to the wild type. *R *is the relative division rate, SFL and SFS are the long-range and short-range swarming fitnesses calculated according to **Eqn 3 and 4**, respectively. **A) **Population size; **B) **Speed of front advancement; **C) **The phases of a simulation experiment (schematic sketch). **D) **The initial phase of population growth (example).

The *in silico *model makes it possible to follow the kinetics of cell populations during the simulation. Swarming experiments show typical saturation-type kinetics (Figure 7) that can be described in terms of approximate initial, transient and steady-state phases. Using the numerical values extracted from the modeling experiments, we can define the swarming fitness of a bacterium (see methods). Briefly, the swarming fitness of a bacterium is proportional to its population size and with the speed of the front advancement. As both of these quantities are in arbitrary units of the model, it is more appropriate to calculate a relative swarming fitness in comparison with a reference, such as the wild type. Using this relative fitness measure, one can construct models that grow faster or slower than the wild type. Figure 7D shows the competition of such models. As can be expected, the fitter (faster growing) model simply outcompetes less fit (slower growing) model. It is also worthwhile to note that nutrients, signals (information) and secreted factors (public goods) are asymmetrically distributed within the activation zone (Figure 2, **inset**), i.e. some parts of the activation zone will be less favorable for growth than others. In accordance with this, we see the less fit cells accumulating in regions less abundant in nutrients and public goods (**insets 1 and 2 in **Figure 7D).

**Figure 8:**
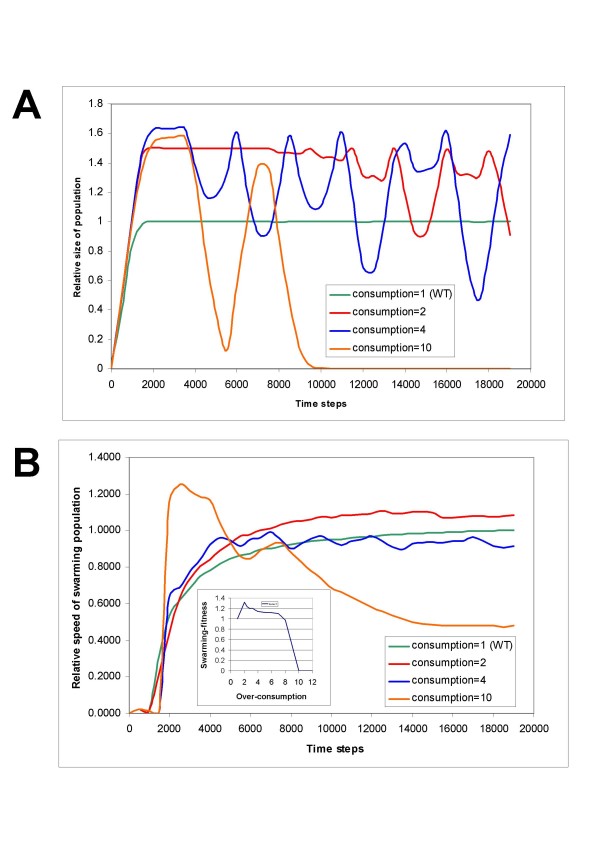
**The effect of over-consumption on the swarming population**. The effect of over-consumption on the swarming population. **A) **Relative population size (compared to that of WT model);**B) **Relative speed (compared to that of WT model). Over-consumption is expressed as nutrient-intake divided by that of the WT. The long range swarming fitness is plotted as a function of over-consumption in the **inset in B**).

In our model system the cells are maintained by a flux of nutrients provided by diffusion. In other terms, their survival depends on a balance between nutrient consumption and diffusion. We can break this balance in two different ways: **a) **by decreasing the flux of nutrients (i.e. decreasing the nutrient concentration or decreasing the diffusion constant of the nutrients), or **b) **making the cells over-consume nutrients. Figure 8 shows a model of the latter strategy that leads to a collapse of the swarming population. As we increase nutrient intake to 10 fold as compared to the WT model, population size decreases 4 orders of magnitude, and the migration slows down. The kinetics shows increasing fluctuations – also seen in a variety of other, non-biological model systems – that finally leads to collapse. The long-range swarming fitness of the population (**Eqn. 3**) first increases with over-consumption but after a limit it decreases to zero (Figure 8, **inset**). It is important to note that the steady population in our longitudinal model corresponds to a colony that steadily grows in two dimensions. In other terms, the model predicts a steady, i.e. sustainable colony growth that can however be collapsed by overconsumption.

## Discussion

In this work, we describe a simple, minimalist model suitable to study the QS behavior of the rice-colonizing bacterium *P. aeruginosa *PUPa3. In this model, in which the bacteria move randomly and the intensity of this motion is under the control of two diffusible materials, the QS signal S and the secreted factor F. The model describes the fundamental properties of QS bacteria, such as **i) **density-dependent onset of the colony-movement, **ii) **displacement towards nutrients. This is achieved without the explicit use of concentration gradients and (hypothetical) chemotactic factors.

Another goal of this study was to gain insight into the kinetics of the computational model. We found that the populations follow a simple, saturation-type kinetics from which one can extract the parameters necessary to define a swarming fitness, the property of a cell to reach a given location by swarming. Models with higher swarming fitness were found to outcompete their less fit counterparts.

A characteristic feature of our model is the transition from a small, completely random population to an "organized" (i.e. asymmetrically distributed) swarming population. This swarming population can be described as an active-zone, in which there is high density of cells, and nutrients, signals and secreted factors are present in concentrations sufficient to support swarming. The active zone is asymmetrical, i.e. cell density as well as chemical concentrations are not uniformly distributed within the zone.

We were explicitly interested in comparing the behavior of the rice-rhizosphere-colonizing bacterium *P. aeruginosa *PUPa3 with its mutants, where either the production of the AHL quorum sensing signals (SN) or the ability to respond to the signal was inactivated (SB). We found that the swarming behavior of the mutants is drastically different from the parent wild type, with neither SN nor SB being able to swarm. However, SN will swarm on agar plates to which the AHL signal molecules have been exogenously added. This behavior confirms that the regulation of swarming by AHL QS in environmental strain PUPa3 is comparable to the one observed in clinical isolates of *P. aeruginosa*, moreover the behavior was reproduced by our minimalist *in silico *model.

The major differences between our model and the previous computational approaches can be summarized as follows: **a) **we are interested in the early stages of swarming, where the functions of a few, key QS genes and gene products determine cell behavior; **b) **instead of studying patterns of colony morphology, our goal is to study the onset of swarming in terms of population size and speed of advancement; **c) **we study this phenomenon using a specific biological object, the rhizosphere colonizer *P. aeruginosa *PUPa3 and its engineered mutants; **d) **the computational model is based on individual cells with threshold-based response behavior. This highly simplified regime was chosen partly because we are interested in the qualitative behavior of the system at the onset of QS (swarming on or off, migration fast of slow), and partly because of the speed of the computation; **e**) the cell-agents move randomly, without being influenced by concentration gradients and by the movements of other cells; **f) **we use a longitudinal plate-arrangement wherein the movement of the front can be followed along one dimension. The small size of the longitudinal track allows one to use accurate, computationally-intensive methods for calculating diffusion. On the other hand, our model can also reproduce branched patterns (not shown), but the longitudinal track allows us to calculate speed values in a computationally more efficient manner.

## Conclusion

We presented a simple model for the swarming of *P. aeruginosa*, wherein the random motion of individual cells is under a threshold-dependent on/off regulation by AHL signals and secreted factors (public goods). In this model, the cellular models are not endowed with self-orienting abilities; however, they self-organize into a community that is capable of spontaneously following environmental signals. The model gave a qualitatively correct description of the behavior of *P. aeruginosa *PUPa3 as well as derivative genomic knock-out mutants, in which either signal production (SN) or signal-response (SB) were inactivated. The experimental results confirm at the same time that QS regulation in the rhizosphere colonizer PUPa3 strain is similar to that observed in the clinical isolates of *P. aeruginosa*.

## Methods

### Bacterial strains and growth conditions

The *P. aeruginosa *strain PUPa3 used in this study is an environmental rice rhizosphere isolate from India [21]. In order to construct the signal negative (SN) mutant of strain PUPa3, both *lasI *and *rhlI *were inactivated via a two-step insertional inactivation using suicide plasmids (Steindler et al., submitted). Similarly, the signal blind (SB) mutant was constructed by inactivating both the *lasR *and *rhlR *genes in strain PUPa3 by insertional inactivation using suicide plasmids (Steindler et al., submitted).

Swarming assays were performed using M8 medium plates (M9 salts without NH_4_Cl) (Kohler et al., 2000) containing 0.5% agar and supplemented with 0.2% glucose and 0.05% glutamate (Murray and Kazmierczak, 2006). The inoculation was performed with a sterile toothpick dipped in a bacterial suspension of OD_600 _2.7. Next, plates were incubated at 30°C overnight, followed by room temperature incubation for additional 48 hours. AHLs were either acquired from Fluka-Sigma-Aldrich or from P. Williams (University of Nottingham, UK) and added exogenously to swarming plates to a final concentration of 2 μM. *P. aeruginosa *was also grown in LB rich media (Sambrook et al., 1989) with 0.5 w/v of agar.

### Description of the computational model

We designed an agent-based model for representing the cells of *P. aeruginosa*. In this model, each cell is an autonomous agent that regulates its own behavior depending on the concentration of nutrients as well as AHL signals (S, F) found in its environment. The cells perform random movements on the 2D plane, and interact with each other via AHL diffusible signals. Each parameter in this system is defined in arbitrary units (further details are given in the Additional file 1).

**Figure 9:**
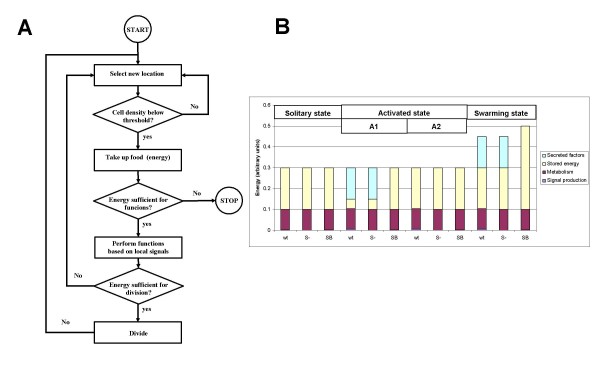
**Properties of cell agents**. **A) **Description of the algorithm carried out by the agents at each time point. At 'STOP', the cells irreversibly enter a metabolically inactive stationary state. The functions depend on the state of activation of each mutant and these are regulated in a threshold-based manner by the signal levels. **B) **Energy balance of the various mutants studied. The numerical values are shown in Table 1 and described in the Additional file 1. "Swarming state" includes two states (with S production "on" [swarming level] or "off" [solitary level]) (Table 1).

Each autonomous agent carries out a simple algorithm (Figure 9A). The functions performed by the cell are regulated in a threshold-based manner according to the regulatory scheme shown in Figure 1A. In the solitary or planktonic state (**P**), there is a baseline level of signal *S *production. As the environmental concentration of *S *exceeds the threshold, the production of *S *increases 5–15-fold (V.V, unpublished results on P. aeruginosa PUPa3), and the production of secreted factor (*F*) starts. This is the activated state (**A**). As soon as the concentration of *F *in the environment surpasses a threshold, the cells increase their nutrient intake and move faster, resulting in the swarming state (**SW**). It can be conceived that the level of S falls below threshold while F is still above it. In this case, the cells move and metabolize at the rate of the swarming state, but production of S falls back to the lower level.

The process is governed by the energy balance of the cells (Figure 9B). At each step, the cells take up a certain amount of nutrients, defined in arbitrary energy units. The energy is spent on maintenance ("metabolism"), production of *S *and *F*, while the remainder is stored. A cell's stored energy can thus be described as

(1) *E*(*t *+ 1) = *E*(*t*) + *E*(*food*) - *E*(*S*) - *E*(*F*) - *E*(*metabolism*),

where *E(t) *is the stored energy at time *t*, while the other terms represent the energy expenditure corresponding to nutrient intake, AHL signal production, nutrient production and metabolism, respectively. If the stored energy exceeds a threshold, the cell divides. If the stored energy is not sufficient to cover the expenditures, the cell will enter into a stationary phase i.e. it irreversibly ceases to function. The agents proceed via random steps, where a step of a given length is taken in a randomly chosen direction. In the swarming state the cells move approximately 3 times faster than in other (solitary or activated) states.

### Diffusing materials

Initially, the environment is represented in terms of a single diffusible material *N*, denoting all nutrients. In the process of the simulation, cells will produce other diffusible materials, such as signal *S *and factor *F*. The concentration of such a component *u *is described by the equation:

(2) $$\frac{du}{dt}=D\nabla^{2}u-Ru,$$

where *D *and *R *are the uniform diffusion and decay constants, respectively. We assume that nutrients diffuse but do not decay (R [nutrients] = 0), but *S *and *F *both diffuse and decay. Equation [2] is a typical reaction-diffusion equation that is solved independently for nutrients and signals on a rectangular grid with periodic boundary conditions using an explicit finite difference method, at each time point of the simulation.

The environment is represented as a 2D longitudinal track with periodic boundary conditions on the longitudinal sides (Figure 2). The plane is discretized into squares, and the concentration of diffusible materials is considered constant within the square. This setup corresponds to a longitudinal cylindrical surface, starting with an impenetrable "wall" at the beginning of the longitudinal "racetrack". At the beginning of the run, the cells have a randomly chosen amount of stored energy, and an equal number of such cells are placed to randomly chosen locations in each square along the starting line. The colony boundary is represented by a line separating the cell colony from the environment. Initially, this separating line will be parallel with the starting line, one square away from it. As the simulation progresses, the cells will move randomly within the boundary while both *F *and *S *diffuse outside the boundary. The advancement of the boundary was modeled according to a modified principle adapted from Cohen [22], that is, the cells' escape attempts were counted for each square of the outer square adjacent to the boundary, by incrementing a boundary advancement counter *BI *with a value of 1+ (k * F), where *F *is the concentration of the factor and *k *is a constant of proportionality. If *BI *reaches a threshold, the border moves past the square in question. As a result, the border advances from square to square.

When compared to models designed to describe colonial patterns, our model is highly simplified, since it is threshold-regulated and does not take into account concentration gradients.

### Evaluation of modeling experiments

We evaluate the results in qualitative terms, and whenever possible, on a comparative basis (e.g. in comparison with the wild type cells. Figure 7 (main text) shows a plot of and population size (Figure 7A) and speed (Figure 7B) as a function of the simulation time (arbitrary units). By way of analogy with reaction kinetics, it is convenient to divide the curves into approximate initial, transient and steady-state phases. As swarming occurs in space, we use the terms "short-range" for the initial phase and "long-range" for the steady-state phase, respectively. The population size and the speed observed in the steady state are independent of the size of the starting population. It is noted that the steady state does not always appear, some model populations (such as the very small populations, or models with inefficient metabolism) die out after a transient swarming phase.

For the characterization of swarming ability, we define swarming fitness as a measure of how efficiently a cell reaches a certain location in space. The swarming fitness of a cell type is proportional to the population size *p *and to the speed *v *observed in the steady state. We can then define the relative swarming fitness of a mutant as

(3) $$SF^{rel}=\frac{p_{m}\times v_{m}}{p_{wt}\times v_{wt}},$$

where the subscripts *m *and *wt *refer to mutant and wild type respectively. For the steady state, p and v values can read from the curves, e.g. we can read averaged values calculated for a longer period of time. The resulting SF value will characterize the mutant's ability to reach a long-range destination. In principle, SF_long_range _is independent of the starting population size, nevertheless we routinely calculated it by using equal starting populations for mutant and wt. The short range swarming fitness, on the other hand, refers to the ability of a cell to reach a destination in an early stage of swarming. Since we can approximately say that the movement of any colony at the onset is very small, so we use the approximation *v*_*m *_≈ *v*_*wt*_. so the short-range relative swarming fitness can be calculated as

(4) $$SF_{short\_range}^{rel}=\frac{p_{m}}{p_{wt}},$$

where *p*_*m *_and *p*_*wt *_indicate the swarming population size taken at a very early time point such as 200 time steps after the onset of swarming (Figure 7C, **inset**). As the short range fitness depends on the initial population sizes so we determined it using strictly identical initial starting populations typically 1000 cells of 2 different models, distributed randomly. Examples of calculated values are shown in the inset in Figure 7C.

Figure 7C and 7D show examples of mutant models that differ in their relative division rates. In our model, the speed of the front movement is mainly regulated by the rate of division, which is, in turn, dependent on the amount of energy that the cells are capable of storing (saving) at each step. Therefore, cells that spend less energy (have less metabolic costs) will be more viable and compete out the cells that spend more energy, as shown by the example in Figure 7D. In these calculations, the rate of division (average division per cell per time point) can be determined directly for the entire experiment by counting the divisions for a given population. The relative division rate, *R*, was then calculated by dividing the division rate of a mutant with that of the wild type. The energy consumption was calculated as *E*(*S*) + *E*(*F*) + *E*(*metabolism*), and the relative energy consumption *E *was calculated by dividing the energy consumption of the mutant with that of the wild type. The energy consumption was then altered so as to produce mutant models with different relative swarming fitness values. The asymmetrical distribution of the two species within the front (**insets 1 and 2 in **Figure 7D) shows that the less successful species is pushed towards the regions containing less nutrients.

## Competing interests

The authors declare that they have no competing interests.

## Authors' contributions

SN designed and implemented the agent-based model and carried out the initial computer modeling experiments and helped draft the manuscript. IB and LS carried out the molecular genetic modifications and the microbiological experiments. ÁK contributed to the computer modeling. VV designed the mutants, while SP designed the modeling framework. VV and SP jointly conceived and designed the study and drafted the manuscript. All authors read and approved the final manuscript.

## Reviewers' comments

### Reviewer 1

#### Gáspár Jékely, Max Planck Institute for Developmental Biology, Tübingen, Germany

**Reviewer's comment: **This paper describes a simple computational model for quorum sensing and a switch to swarming behaviour in *Pseudomonas aeruginosa*. The paper is interesting and well written. The most important finding is that under certain conditions it is possbile to obtain a moving „activation zone" of swarming cells that moves towards nutrients without invoking directional response, such as chemotaxis. My question is whether such conditions are met in real life, in particular the diffusion coefficients used. In the model the activation zone moves in one direction, because the nutrients are depleted behind the front. The extent of such depletion will be influenced by the diffusion constant of the nutrients. If nutrient diffusion is fast with respect to cell division rates and swarming rates, then I suspect that the activation zone will not move towards the source of nutrients and the model fails. I think it would therefore be important to test different diffusion constants for the nutrients and see whether the parameters which are able to maintain directional swarming, using realistic cell doubling times and swarming speed, are near the real values in tissues or agar plates.

**Authors' response: **The suggestion is in line with our modeling experiences. Namely, the population is maintained by the flux of nutrients provided by diffusion i.e. its survival depends on a balance between nutrient consumption and diffusion. We have added a new paragraph and a figure in which we show that breaking this balance abolishes swarming, exactly as the reviewer predicted.

**Reviewer's comment: **The simulations are performed in a 2D longitudinal track (a surface of a slender cylinder), which is almost in 1D. This approach has certainly its merit, especially when measuring the advancement of the active zone. However, if the model is correct, it should also reproduce the dendritic colony morphologies when extended to two dimensions. Using the computational framework such an extension seems to be straightforward, just the modelled area should be increased. I presume that dendritic morphologies develop because cells move randomly and the initiated advancing fronts deplete the nutrients around them.

**Figure 10:**
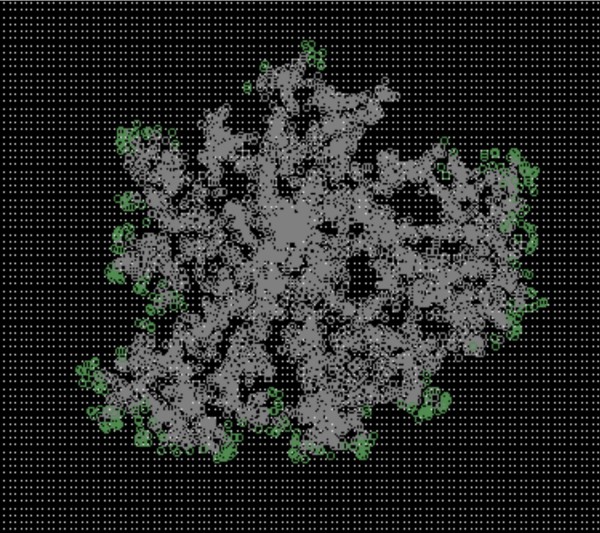
**A dendritic pattern produced the hybrid model**.

**Authors' response: **In fact, the modell can provide dendritic patterns, as shown by Figure 10. We used the 1D arrangement since we are interested in the speed of front movement (fast growth, slow growth, etc.), which is not straightforward to define for uneven, dendritic patterns.

The realism of such dendritic patterns is questionable, however, since, for computational reasons, we can not model large populations of cells such as present on a single agar plate. Moreover, we can suppose that not only a few genes, but a large part of the genome may contribute to such patterns. This is why we thought about limiting our interests and our interpretations to a smaller context, i.e. the onset of swarming.

**Reviewer's comment: **The model assumes a threshold signal concentration to activate the quorum sensing response. Has this been demonstrated experimentally before? If not, it would be easy to test such threshold response using the swarming experiments in the SN mutants, using different concentrations of the signal.

**Authors' response: **The genetic networks underlying QS are in fact considered to act as a two-state switch (Goryachev et al. Transition to quorum sensing in an Agrobacterium population: a stochastic model. PLOS Comp Biol, 1(4), 2006, 265–275). This is a working hypothesis of our minimalist model, and our goal was to simplify the process as much as possible.

We thank the referee for the interesting comments and questions.

### Reviewer 2

#### L. Aravind, National Center for Biotechnology Information, National Library of Medicine, National Institutes of Health, Bethesda, MD 20894

**Reviewer's comment: **Bacterial quorum sensing and swarming are both interesting topics of study in themselves, and also of considerable significance to understand certain behaviors of pathogens. Previously there has been considerable effort in using hybrid models (i.e. cells as stochastically orienting entities and molecular influences as continuous density fluctuations) in describing branching and nested patterns of colonies. Here the authors apply the same to describe swarming in *Pseudomonas aeruginosa*.

The salient features of their model are: 1) Its simplicity allows it to be easily understood and potentially reproduced. 2) It generates a good qualitative match with experimentally observed behavior of the wild-type and that of mutants. 3) Most interestingly they are able obtain the displacement towards environmental cues or nutrients as an emergent property despite starting with cells without self-orienting abilities. That this behavior emerges is something which could be tested experimentally – if the model indeed survives such experimental tests, such models could have a general implication for understanding emergent population behaviors in bacteria. 4) The concept of swarming fitness developed here is a useful construct to directly study genes that might affect this property.

Primary criticisms: For the model to acquire greater biological relevance it would be useful if it had an "internal component" – i.e. the cells are provided with a regulatory network that recapitulates the experimentally well-understood QS network in *Pseudomonas*. Even if it might be too much redo everything with such an internal network model for this work, it would be useful if the authors actually lay out its essential elements here.

**Authors' response: **Our aim is to develop a simplistic, minimalistic model in which the internal genetic network is represented simply as a binary switch. Our main goal is to explore what this minimalist model can do and describe the fundamental properties of the model. This model is not meant to be realistic in any sense, we only seek to reproduce the on/off behavior of fast or slow migration at the onset of swarming. A more detailed genetic network is a different approach that we can consider in later studies.

**Reviewer's comment: **This model with a relatively simple set up explains certain behaviors as emergent without assuming any kind of in-built cell-cell interaction. But is this reflective of the natural situation in a proteobacterium with such a large genome? Cells potentially behave differently towards kin as against non-kin. If such interactions are included do we observe differences (The reason this question comes to mind is due to the hybrid model being used, where such things could be included)?

**Authors' response: **Competition experiments are possible and will be the subject of further, more detailed studies. This is now mentioned in the text.

**Reviewer's comment: **Finally, the question of completeness of such a model for the whole system does come up. The authors look at a 1D element but the experimental results shown to the side are from colony with nested structure. Are the assumptions provided here enough to explain the colony morphology also or do they need additional explanations?

**Authors' response: **In fact, the modell can provide dendritic patterns, as shown by Figure 10. We used the 1D (more exactly: cylindrical) arrangement since we are interested in the speed of front movement (fast growth, slow groth, etc.), which is not so straightforward to accurately calculate for uneven, dendritic patterns

**Reviewer's comment: **Minor points: There are typos/grammatical slips throughout the paper. A thorough proof-reading will improve the article.

**Authors' response: **We made corrections. We wish to thank the reviewer for the interesting questions, comments and suggestions.

### Reviewer 3

#### Eugene V. Koonin, National Center for Biotechnology Information, National Library of Medicine, National Institutes of Health, Bethesda, MD 20894

**Reviewer's comment: **Netotea et al. describe a simple mathematical model of quorum sensing in Pseudomonas in which the behavior of individual bacterial cells is limited to random movements, without the ability of self-orientation but swarming nevertheless occurs as an emerging phenomenon that is predicated on the threshold-dependent response of bacteria to the diffusible quorum signal (AHL) and secreted factors. Notably, it is shown in the same paper that knockout of genes involved in the synthesis or sensing of AHL yields results compatible with the model. This seems to be not just an interesting piece of work but, actually, an important piece of work the result of which, in my opinion, should be taken as the null model for explaining the fascinating behavior of bacteria during quorum sensing.

**Authors' response: **We thank the reviewer for the valuable comments.

### Reviewer 4

#### Artem Novozhilov, National Center for Biotechnology Information, National Library of Medicine, National Institutes of Health, Bethesda, MD 20894 (nominated by Eugene Koonin)

**Reviewer's comment: **In this manuscript S. Netotea et al. provide a simple computational model of the initiation of the bacterial swarming by means of quorum sensing. The model the authors employ is of a hybrid type, which means that they model cells as discrete entities subject to random movement, whereas the diffusion of nutrients, signals and public good are represented as continuous diffusion equations. The authors base their model on an explicit regulation of the bacterial behavior depending on the density of the cells. The main computational findings are illustrated by *in vitro *experiments, which show good qualitative agreement with modeling results. In particular the authors show that the concerted cell movement occurs in their model without bringing in any self-orientation abilities such as, e.g., ability to move on the gradient of a chemotaxic signal. The text is written very clearly, and the authors make good work explaining their algorithm, experiments, major results, and shortcoming of the chosen approach. Overall, I find the subject extremely interesting and the approach is worth pursuing.

My major criticism concerns the fact that, in my view, the model is somewhat oversimplified. In particular, the state of a given cell is defined using explicitly given condition: if the concentration of the signal exceeds some threshold quantity then the production of the signal increases in a deterministic way prescribed by the algorithm (a side question: in your model the production of *S *increases in 5–15 times whereas it is well-accepted that 100-fold increase is a conservative estimate [Goryachev et al. Transition to quorum sensing in an Agrobacterium population: a stochastic model. PLOS Comp Biol, 1(4), 2006, 265–275]?).

**Authors' response: **The picture of quorum sensing has a threshold level of signals is generally accepted in the field (Fuqua et al, Regulation of Gene Expression by Cell-tio-cell communication: Acyl-Homoserine Lactone Quorum Sensing. Annu. Rev. Genet. Volume 35, 439–468) so we accepted it as a working hypothesis. Simplification was one of our main goals as we believe that simplistic (minimalistic) models are useful to explore a core set of properties underlying a particular behavior. To give an example, lattice models of protein folding are not realistic in any way, yet they provided interesting insights. We believe that such models are not designed to be accurate representations of particular realities. Rather they are abstractions designed to represent general and in some cases emergent sets of properties and patterns.

The figure of 5–15 fold increase in signal production is based on our experimental gene expression results obtained on our own laboratory strain. This is now mentioned in the text. Such values seem to depend on the strain and on the conditions.

**Reviewer's comment: **It would be more interesting to model a gene network, which works as a stochastic switch (say, similar to the one, considered in Goryachev et al. Transition to quorum sensing in an Agrobacterium population: a stochastic model. PLOS Comp Biol, 1(4), 2006, 265–275). In this case it is possible to follow two modeling processes at a time (onset of the quorum sensing at the level of i)ndividual cells and the spatial distribution of the cell population). Although I have to admit that this model improvement might be well beyond of the present manuscript.

**Authors' response: **This suggestion will be followed in future studies; our current goal was to determine how a stripped-down, simplistic model behaves.

**Reviewer's comment: **Demonstrated cell swarming can be a consequence of the oversimplifications of the underlying processes. The authors mention in the text that 'A conspicuous feature of our model is the transition from a small, completely random population to an "organized swarming population" '. I do not see any reason why, giving the set up of the model, one could not get this directed movement from the areas of no nutrients to the areas with high nutrients concentration. Yes, indeed, there is no direct incorporation of any mechanism of directed movement in the model, but, how it is well known, to produce traveling wave solutions (what is actually shown in Fig. 2) there is no need to include chemotactic terms in the model. Diffusion (random movement) plus nonlinear reaction terms are enough. In this respect, it would be interesting to consider fully deterministic model, i.e., replace the agent-based modeling of the cell movement by a reaction-diffusion equation. In this case, traveling wave solution can be studied analytically (at least, in principle, I might be too optimistic here).

**Authors' response: **Hybrid models can be analogous to continuum models described by reaction-diffusion equations. However, our goal was to set up a hybrid model in which the behavior of individual cells can be studied and compared with agar plate experiments. The construction and characterization of a continuum model for this system is a project yet to be done which is, in our view, outside of the immediate scope of the present work. But the continuum models are in fact worth to study.

**Reviewer's comment: **And this brings in the second criticism of the text. The modeling experiments are such that can be done with the model, which is fully continuous. The potential of the agent-based modeling is not taken advantage of. The main advantage of the agent-based modeling is an ease to include cell heterogeneity in the model. There is still no generally recognized opinion that quorum sensing *per se *(i.e., the reaction for the cell density) is the mechanism responsible for the concerted cell behavior. There is an alternative explanation: diffusion sensing (and what is actually modeled in the text, because the decision to increase signal production is made on the signal concentration, which diffuses in space and not on the cell density). The major difference between quorum sensing and diffusion sensing comes from evolutionary point of view: quorum sensing is a collective property, whereas diffusion sensing is an individual property. To explain the evolution of the former one needs to explain the evolution at the group level and also give an explanation why cheaters (the cells that make other cells to produce public goods but do not spend their energy to produce them by themselves) do not invade the population (more on this in Hense et al. Does efficiency sensing unify diffusion and quorum sensing? Nature Reviews Microbiology, Volume 5, 2007, 230–239). I would suggest setting the model experiment when the cell population is heterogeneous; there are altruists and cheaters, and consider the following question: can, actually, swarming be a mechanism that allows survival of the altruists (it is well known that the explicit spatial structure often mediates survival of altruists).

**Authors' response: **In our system, diffusible materials are the only means of communication, so cell-agents "sense" elevated cell density via the accumulation of these. This simple setup is apparently sufficient for the agents to follow exogenous signals and form asymmetrically distributed populations. Our interpretations do not go beyond this point. The suggestion of studying competing cell populations is in fact a challenging task for the future, and, the reviewer is right, this model was actually chosen with a perspective of studying such, more complex systems. However, we wanted to limit the scope of the present work to the fundamental properties of the model. An example of competition experiments is actually shown in Figure 7D.

**Reviewer's comment: **It is also not quite clear why the authors decided to model actually 1D spatial structure, because it seems to be straightforward to implement the same algorithm, starting from the center of some 2D area. In this case the comparison of *in vitro *and *in silico *experiments will be simpler. The obvious question is whether it is possible to observe in the model the fractal structures as in Figure 5 and 6 on swarming plates.

**Authors' response: **Our goal was to study the speed of movement and this is more straightforward and also computationally much more efficient in a small system (we mention that, the system is not exactly 1D, the agents migrate on an open-ended cylindrical surface). On the other hand, our model can produce dendritic growth patterns (Figure 10), but the modeling of vast cell populations such as observed on agar plates, is outside our computational limits.

We wish to thank the reviewer for the interesting and challenging questions that, at least in our opinion, helped a great deal to improve our manuscript.

## Supplementary Material

Additional File 1 **Appendix.** Parameters used in the computational model. Click here for file
