# Supplementary material for: A simple model for the early events of quorum sensing in Pseudomonas aeruginosa: modeling bacterial swarming as the movement of an "activation zone"
Source: Biol Direct. 2009 Feb 12;4:6. doi: 10.1186/1745-6150-4-6 (PMC2660287; doi:10.1186/1745-6150-4-6)
Supplement: Additional File 1 — Appendix. Parameters used in the computational model. [file 1745-6150-4-6-S1.pdf]

Appendix: Parameters used in the computational model

Netotea et al. A simple model for the early events of quorum sensing in *Pseudomonas aeruginosa*: modeling bacterial swarming as the movement of an "activation zone", submitted to Biology Direct, 2008

Global parameters

Parameter	Typical value
Medium size X	250[a]
Medium size Y	2000[a]
Lattice square size	5[a]
Lattice size X	50 squares
Lattice size Y	400 squares
Initial nutrient per square	2000[b]
Initial signal per square	0[c]
Maximum number of bacteria per square	10

Values given in arbitrary units; [a]length units, [b]nutrient concentration units, [c]signal concentration units

Movement parameters

Parameter	Typical value
Cell movement/time point	
Solitary and activated states	1.5[a]
Swarming states	5[a]
Border movement	
Border advancement threshold	320
Border advancement constant *k*	5

Energy expenditure per time point

	Solitary state (S off, F off)			Activated state A1 (S on, F off)			Activated state A2 (S off, F on)			Swarming state (S on, F on)		
	WT	SN	SB	WT	SN	SB	WT	SN	SB	WT	SN	SB
**Signal production**	0.001	0.000	0.001	0.005	0.000	0.001	0.005	0.000	0.001	0.005	0.000	0.001
**Metabolism**	0.100	0.100	0.100	0.100	0.100	0.100	0.100	0.100	0.100	0.100	0.100	0.100
**Stored energy**	0.199	0.200	0.199	0.045	0.050	0.199	0.195	0.200	0.199	0.195	0.200	0.349
**Secreted factors**	0.000	0.000	0.000	0.150	0.150	0.000	0.000	0.000	0.000	0.150	0.150	0.000
**Nutrient intake**	0.300	0.300	0.300	0.300	0.300	0.300	0.300	0.300	0.300	0.450	0.450	0.450
**Total energy consumed**	0.101	0.100	0.101	0.255	0.250	0.101	0.105	0.100	0.101	0.255	0.250	0.101

Off and ON = value below and above threshold, respectively. Values are expressed in arbitrary energy units and indicate typical parameter settings for each time point. The threshold for division was expressed in the same units and typically set to 12.0.

Secreted products

	Production/cell/time-point	Threshold
Signal S	0.5	10
Factor F	0.1	10

All values are given in arbitrary units

Diffusion and decay parameters

Name	Signal *S*	Factors *F*	Nutrients *N*
Diffusion coefficient *D*	0.02	0.05	0.03
Decay rate *R*	0.001	0.0001	0.0

All values are given in arbitrary units
